# Imatinib-Associated Tumor Lysis Syndrome in a Patient With Myeloid Neoplasm With Eosinophilia and *PDGFRA* Rearrangement: A Case Report and Review of the Literature

**DOI:** 10.1200/JGO.2016.007716

**Published:** 2017-02-08

**Authors:** Francisco Socola, Veronica Hawes, Rodolfo Henrich Lobo, Appalanaidu Sasapu

**Affiliations:** All authors: University of Arkansas for Medical Sciences, Little Rock, AR.

## INTRODUCTION

The term hypereosinophilic syndrome (HES) is defined as a persistent elevation of the eosinophil count ≥ 1,500/mm^3^ in the peripheral blood for at least 6 months, with evidence of end-organ damage.^1^ Etiologies for some forms of HES have been described, and the 2016 revision to the WHO classification of myeloid neoplasms and acute leukemia separates neoplasms associated with hypereosinophilia into two distinct groups. The first group falls under the category of myeloproliferative neoplasms, and it is named chronic eosinophilic leukemia, not otherwise specified. It is characterized by an eosinophil count ≥ 1,500/mm^3^, with evidence of clonality in the eosinophil lineage, or an increase in myeloblasts, but < 20%, in peripheral blood or bone marrow. In the absence of clonality or increased blasts, the diagnosis of idiopathic HES is made. By definition, there should be no Philadelphia chromosome or a rearrangement involving *PDGFRA/B* and *FGFR1*^2^. The second group falls under the category of myeloid/lymphoid neoplasms with eosinophilia and rearrangement of *PDGFRA*, *PDGFRB*, or *FGFR1*, or with *PCM1-JAK2*.^3^ The most common molecular abnormality is rearrangement of *PDGFRA*, and it predicts a favorable response to imatinib; however, the incidence of *FIP1L1-PDGFRA* rearrangement in patients with hypereosinophilia is only 10% to 20%.^4,5^

Given the historically poor prognosis of chronic eosinophilic leukemias and the exquisite sensitivity to imatinib in patients with rearranged *PDGFRA/B*, consensus has emerged that these individuals be treated even in the absence of organ dysfunction.^6^ Several case reports and small case series of patients with chronic eosinophilic leukemia with *PDGFRA/B* rearrangement have described the efficacy of imatinib at a dose of 100 to 400 mg per day to produce durable complete hematologic responses.^7-11^

## CASE REPORT

**Fig 1:**
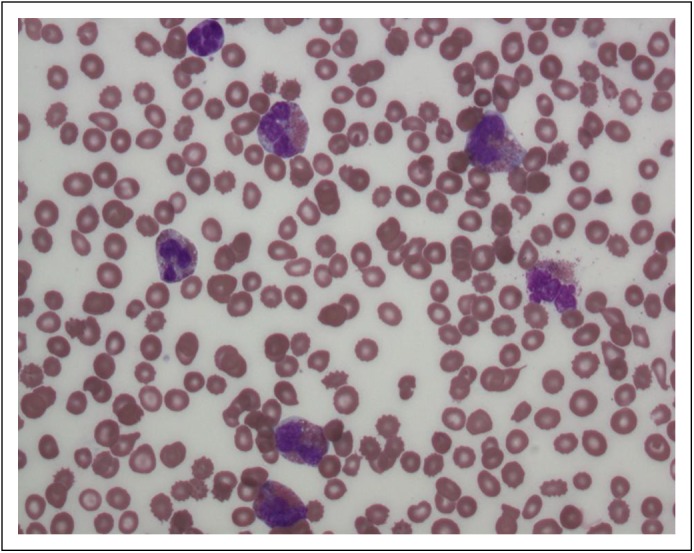
– Peripheral blood smear showing morphologically abnormal eosinophils. Note the large, bright-orange granules.

**Fig 2:**
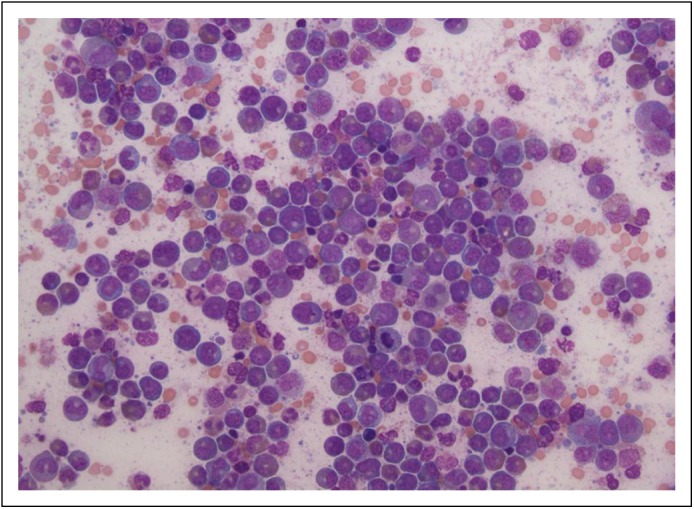
– Bone marrow aspirate with a marked increase in eosinophils and eosinophilic precursors, admixed with approximately 8% blasts.

A 60-year-old African American man with a medical history of hypertension, alcoholic cirrhosis, chronic obstructive pulmonary disease, and recent diagnosis of chronic eosinophilic leukemia (CEL) was transferred to our hospital for fever, elevated WBC count of 61,000/mm^3^, and urinary tract infection symptoms. Two months earlier, he was admitted to an outside hospital with severe fatigue, low appetite, and weight loss. During that admission, he was found to have an elevated WBC count of 50,000/mm^3^, with a differential showing 22% neutrophils, 21% lymphocytes, 46% eosinophils, and 2% basophils. The hemoglobin level was 9.1 g/L, and the platelet level was 70,000/mm^3^ (Fig 1). Bone marrow examination performed at the referring institution revealed a markedly hypercellular bone marrow (100% cellularity), involved by a myeloid neoplasm with marked eosinophilia and approximately 15% CD34-positive blasts on core sections; a standard differential performed on the aspirate smears revealed approximately 8% blasts. In addition, it showed marked myeloid hyperplasia, including an increased number of atypical eosinophils, dysgranulopoiesis, and fewer erythroid precursors (Fig 2). Flow cytometric analysis performed on the aspirate revealed increased myeloblasts (11% of total events) and a marked increase of eosinophils (54%). Fluorescent in situ hybridization testing was significant for a translocation of *PDGFRA/4q12*, and metaphase cytogenetics revealed an abnormal male karyotype, 46,Y,t(X;5)(p11.4;p15.3)[6]/46,XY.^4^ Additional fluorescent in situ hybridization studies performed at our institution on a subsequent bone marrow examination confirmed the *PDGFRA* rearrangement; in addition, the recurrent cytogenetic abnormalities associated with acute myeloid leukemia t(15;17), t(8;21), and inv(16) were all excluded. Reportedly, the patient started receiving oral imatinib 400 mg daily at the outside hospital for 3 to 4 weeks, but it had to be discontinued because of severe pancytopenia. We reviewed all the outside hospital hematology notes to learn why the patient was receiving oral imatinib 400 mg daily; however we could not find the answer to this question.

On arrival at our hospital, the patient was confused, afebrile, and tachycardic, with physical examination notable for temporal wasting, 3+ pitting edema in his feet bilaterally, a distended but nontender abdomen with splenomegaly, and a stage 3 sacral decubitus ulcer. There were no palpable enlarged lymph nodes or skin rashes. The patient denied nausea, vomiting, diarrhea, constipation, easy bleeding or bruises, abdominal pain, and fevers. The presenting CBC and chemistries were as follows: WBC, 59,000/mm^3^ with marked eosinophilia; hemoglobin, 9 g/L; platelet count, 11 × 10^3^/mm^3^; creatinine, 1 mg/dL; potassium, 3.8 mEq/L; sodium, 139 mEq/L; blood urea nitrogen,18 mg/dL; phosphorus, 2.1 mg/dL; and calcium, 7.8 mg/dL. A complete abdominal ultrasound revealed moderate splenomegaly, with a calculated splenic volume of 1,318 mL, a moderate amount of ascites, and normal sonographic appearance of the liver without intrahepatic or extrahepatic ductal dilation.

Blood and urine cultures were taken, a platelet transfusion was given, and the patient was given intravenous ceftriaxone and fluids because the urine culture taken 3 days earlier at the outside hospital revealed *Klebsiella* species. Once the patient improved clinically, he received imatinib 400 mg daily by mouth for his myeloid neoplasm with eosinophilia and *PDGFRA* rearrangement. After 2 days of imatinib treatment, the WBC count decreased to 36,000/mm^3^, the creatinine level went up to 1.6 mg/dL, the uric acid level was 14.4 mg/dL, the potassium level was 6 mEq/L, the phosphorus level was 7.1 mg/dL, the calcium level was 7.2 mg/dL, and the lactate dehydrogenase level was 369 U/L. These laboratory abnormalities were compatible with tumor lysis syndrome (TLS), and the patient was given rasburicase (Elitek; Sanofi, Brightwater, NJ), allopurinol (Zyloprim; Prometheus Laboratories, San Diego, CA), sodium polystyrene sulfonate (Kayexalate; Sanofi), and aggressive hydration (Table 1). Two days later, the creatinine level decreased to 0.9 mg/dL, the uric acid level decreased to 7.6 mg/dL, the lactate dehydrogenase level normalized, and the WBC count decreased to 5,000/mm^3^. Imatinib was decreased to 200 mg daily. The patient stayed in the hospital for 2 weeks for treatment of his acute urinary tract infection, up-titration of furosemide and spironolactone, and stabilization of his portal hypertension. He tolerated the low-dose imatinib well without requiring blood transfusions.

**Table 1:**
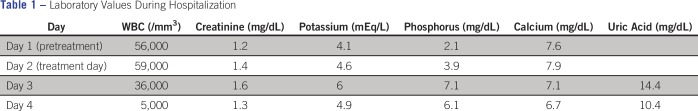
– Laboratory Values During Hospitalization

## DISCUSSION

To the best of our knowledge, this is the first reported case of chronic eosinophilic leukemia with *PDGFRA* rearrangement associated with TLS as a complication of imatinib therapy. We found only one case in the literature that reported TLS as a complication of imatinib treatment of HES without *PDGFRA* rearrangement. That patient was an 83-year-old woman who presented with an elevated WBC count (186,300/mm^3^), with eosinophils > 90%. The eosinophils expressed CD7/13/33/34/DR, and the karyotype demonstrated 47,XX,+8. Results of testing for fusion gene of *FIP1L1*/*PDGFRA* in peripheral blood were negative. The patient was treated initially with prednisolone and hydroxyurea, with limited effect; then, corticosteroid pulse and imatinib (100 mg/day) were administered. A prompt response was observed, with a rapid decline in WBC count, but TLS led to acute renal failure and disseminated intravascular coagulation. Despite aggressive supportive therapies with dialysis and transfusions, the patient died as a result of alveolar hemorrhage.^12^

To have a better knowledge of the possible adverse effects of imatinib in this disease, we analyzed the results of two prospective studies that investigated the efficacy of this tyrosine kinase inhibitor at different doses. In 2007, Baccarani et al^10^ reported the results of a prospective multicenter study of 27 patients with CEL with *PDGFRA* rearrangement who were treated with imatinib, beginning with 100 mg daily for 1 week. Thereafter, the daily dose was increased by 100 mg each week and was set at 400 mg from week 4 on. Imatinib treatment was continued for a minimum of 4 weeks in the case of no hematologic response or until it was beneficial for the patient in case of response. During year 1, the dose was adjusted for toxicity and adverse events, according to the standard criteria for dose adjustment used in the treatment of chronic myeloid leukemia. In this study, all 27 patients achieved a complete hematologic response within 1 month, and all of them remained in continuous complete hematologic response until last contact, with a median follow-up of 25 months (range, 15 to 60 months). At the end of the study, all patients were still receiving imatinib at a daily dose of 400 mg (eight patients), 300 mg (two patients), 200 mg (10 patients), and 100 mg (seven patients). The most common hematologic adverse effects were grade 2 neutropenia in 7.9% of patients and grade 3 neutropenia in 3.2% of patients; the most common nonhematologic adverse events consisted mainly of myalgia and muscle cramps (9.5% grade 2 and 3.1% grade 3) and diarrhea (6.3% grade 2), but also skin rash, abdominal pain, edema, headache and paresthesia.^10^ TLS was not reported as a possible adverse effect in this study. Jovanovic et al reported the polymerase chain reaction results of 17 patients with CEL and *PDGFRA* rearrangement treated with imatinib, the patients were given up-front daily doses of 100, 200, 300, and 400 mg. They found that 11 of 11 evaluable patients achieved at least a 3-log reduction in *FIP1L1*-*PDGFRA* fusion transcripts relative to the pretreatment level within 12 months; interestingly, four of these patients started receiving a daily dose of 400 mg of imatinib, and none of them were reported to have TLS.^11^

In conclusion, to our knowledge, we report the first case of a patient with CEL with *PDGFRA* rearrangement associated with TLS as a complication of imatinib therapy. We think the most likely contributing factors were the high starting dose of imatinib (400 mg daily) and the elevated WBC with high blast count. We want to emphasize that the US Food and Drug Administration–recommended starting dose for patients with the *FIP1L1-PDGFRA* rearrangement is 100 mg daily. Cumulative data with long-term follow-up indicate that this dose is sufficient to elicit complete and durable hematologic and molecular remissions. For patients with myeloid neoplasms (usually myelodysplastic syndrome/myeloproliferative neoplasms) with eosinophilia and rearranged *PDGFRB*, the recommended imatinib dose is 400 mg daily, which reflects the dose consistently used in several case series with excellent outcomes.^6^
